# Exploring motivational patterns in high-performing pianists: evidence from Cliburn competitors’ biographies

**DOI:** 10.3389/fpsyg.2024.1426235

**Published:** 2024-10-18

**Authors:** Xiao Chen

**Affiliations:** McDougall Faculty of Business, University of Prince Edward Island, Charlottetown, PE, Canada

**Keywords:** implicit motives, motivational pattern, the Cliburn, biographies, linguistic inquiry and word count

## Abstract

This research examines the motivational patterns of high-performing classical pianists, characterized by a combination of implicit motives (i.e., non-conscious preferences for specific incentives). Utilizing the Linguistic Inquiry and Word Count (LIWC) software, I analyzed textual data from biographies of 107 pianists (i.e., Juniors aged 13–17: *n* = 38; Professionals aged 18–30: *n* = 30; Amateurs aged 35 and older: *n* = 39) participating in the prestigious 2022–2023 Van Cliburn Competitions. My results showed distinct profiles of implicit motives among pianists compared to non-pianists, with significantly higher need for achievement and need for power. While professional pianists exhibited the lowest level of need for power, junior pianists demonstrated the highest level of need for affiliation. Gender and age predicted part of pianists’ implicit motives. Male pianists demonstrated higher need for achievement than females. Finally, age negatively predicted need for affiliation. These findings highlight the motivational patterns within the classical piano community, offering theoretical implications for understanding implicit motives and practical applications for pianist education. Study limitations and future research directions are discussed.

## 1 Introduction

Pianists hold a pivotal position within the classical music sphere, serving not only as performers but also as collaborators (e.g., Katz, 2009) and educators (e.g., Duckworth, 1965). Their contributions extend beyond the concert hall, shaping the cultural landscape and fostering musical appreciation worldwide. While previous research has explored the psychological profile of high-achieving pianists (e.g., NEO Five-Factor in Chmurzynska, 2012), less attention has been paid to the deeper, motivational drivers that underpin pianists’ aspirations, behaviors, and actions.

Understanding pianists’ underlying motivation is crucial for several reasons. Firstly, it allows us to gain insight into the driving forces behind their dedication to their pianistic craft, shedding light on the intrinsic and extrinsic factors that fuel their pursuit of musical excellence (*cf.* Amabile, 1996). Secondly, insights into pianists’ motivations can inform pedagogical approaches (Gerelus et al., 2020), career development strategies (Zhukov and Rowley, 2022), and performance enhancement techniques (Chaffin and Lemieux, 2004; Osborne et al., 2020), ultimately benefiting both individual pianists and the broader classical music community. Furthermore, understanding pianists’ motivational dynamics can facilitate the cultivation of supportive environments that nurture their artistic growth and well-being (Koehler et al., 2023), thereby ensuring the continued vitality and sustainability of the classical music ecosystem.

Despite the significance of understanding pianists’ motivational profiles, there persists a notable gap in our understanding of their implicit motives (e.g., McClelland, 1987; Schultheiss and Brunstein, 2010). Operating outside an individual’s conscious awareness, implicit motives are motivational predispositions based on affective responses to rewards and punishments that facilitate the pursuit of specific types of incentives and the avoidance of certain disincentives (Schultheiss and Köllner, 2021). Studying implicit motives can provide distinctive insights into individuals’ genuine passions, values, and enduring aspirations, which may not always align with their explicitly stated goals or objectives (Köllner and Schultheiss, 2014; Rawolle et al., 2013; Schultheiss and Köllner, 2021).

### 1.1 Implicit motives

David McClelland’s Implicit Motives Theory (McClelland, 1987; Schultheiss and Brunstein, 2010) posits that implicit motives direct and drive individuals’ behavior towards emotionally charged or affectively “hot” stimuli (Schultheiss, 2008; Schultheiss et al., 2010). Since the 1950s, research on implicit motives has predominantly focused on “big three” fundamental motivational needs: need for achievement (*n* Achievement), need for power (*n* Power), and need for affiliation (*n* Affiliation). Below, I outline the definitions of these motives, their psychological and behavioral correlates (see Schultheiss and Köllner, 2021, for a recent comprehensive review), and their relevance to pianists.

#### 1.1.1 Need for achievement

Need for achievement is “the capacity to derive satisfaction from the autonomous mastery of challenging tasks” (Schultheiss and Köllner, 2021, p. 385). *n* Achievement drives individuals to excel in challenging tasks and seek personal accomplishment (McClelland et al., 1953). Understanding pianists’ level of *n* Achievement can provide insights into their drive for mastery (Schultheiss and Brunstein, 2005), goal-setting behaviors (Brunstein, 2010), and responses to feedback (Brunstein and Maier, 2005). Pianists with high *n* Achievement may demonstrate a preference for challenging repertoire, set ambitious performance goals (e.g., Yuja Wang’s 2023^1^ marathon Carnegie concerts featuring Rachmaninoff’s five works for piano and orchestra), and persist in the face of setbacks (Neumann and Schultheiss, 2015). In contrast, those with lower *n* Achievement may exhibit less ambition and persistence in their musical pursuits (McClelland, 1987; Schultheiss and Brunstein, 2010). By understanding *n* Achievement among pianists, piano educators and researchers can tailor training and support interventions to enhance achievement motivation and facilitate their artistic development.

#### 1.1.2 Need for power

Need for power refers to “a capacity to derive pleasure from having physical, mental, or emotional impact on other individuals or groups of individuals and to experience the impact of others on themselves as aversive” (Schultheiss and Köllner, 2021, p. 387). *n* Power predicts an individual’s assertiveness (e.g., Veroff, 1982), leadership (e.g., Steinmann et al., 2015), and competitiveness (e.g., Winter, 2010). Pianists with a high *n* Power may be more inclined to seek opportunities for solo performance, assert their artistic vision, and engage in competitive contexts such as competitions and auditions. Understanding the interplay between *n* Power and musical outcomes can inform strategies for fostering healthy competition, enhancing leadership skills, and navigating interpersonal dynamics within collaborative settings (*cf.* Ditlmann et al., 2017).

#### 1.1.3 Need for affiliation

Need for affiliation is “a capacity to derive satisfaction from establishing, maintaining, and restoring positive relationships with others and to experience separation as aversive” (Schultheiss and Köllner, 2021, p. 386). *n* Affiliation is related to interpersonal relationships, social connections, and collaborative behaviors (e.g., Dufner et al., 2015). Pianists with a high *n* Affiliation may prioritize building supportive networks (e.g., Dufner et al., 2018), seek opportunities for ensemble playing, and thrive in cooperative musical environments. By contrast, those with lower *n* Affiliation may gravitate towards solo performance or struggle with interpersonal challenges in ensemble settings. By understanding the role of *n* Affiliation among pianists, pianist educators and researchers can develop interventions to promote positive social interactions, foster collaborative skills, and enhance the sense of community within the classical music scene.

### 1.2 Research questions

Exploring each of McClelland’s implicit motives among pianists offers valuable insights into their motivational profiles. Previous research on implicit motives has highlighted that effective leaders often exhibit a specific motivational pattern known as the “leadership motive pattern” (LMP) (McClelland and Boyatzis, 1982; McClelland and Bunham, 1976; Winter, 1973; Winter, 1991). The LMP is characterized by a combination of high *n* Power, low *n* Affiliation, and a high level of activity inhibition (AI), or “a stable tendency to refine and regulate the behavioral expression of motives” (Steinmann et al., 2015, p. 168). In this study, I aim to explore whether high-performing Cliburn pianists possess a distinct combination of implicit motives, which I refer to as a musicianship motivational pattern (MMP). Consequently, I focus on three primary research questions (RQs):

RQ1: *How do the implicit motives of high-performing pianists compare to those of non-pianists*?

RQ2: *Do the implicit motives of high-performing pianists differ across distinct competitive settings*?

RQ3: *How do pianists’ sociodemographic* var*iables, such as gender and age, relate to their implicit motives*?

## 2 Method

### 2.1 Research setting: the Van Cliburn competition (2022–2023)

The Van Cliburn Competition offers a rich and multifaceted research context for understanding the motivational profiles of high-performing pianists. As one of the most prestigious piano competitions globally, it attracts top-tier pianists from around the world, ensuring a pool of participants who have demonstrated exceptional dedication and talent in their pursuit of piano mastery. This concentration of elite performers provides a unique research context to study individuals who have already achieved a high level of musical success and competence, offering valuable insights into the motivational factors driving drive their continued pursuit of excellence.

The competitive nature of the Cliburn Competition replicates the high-stakes performance environments that professional pianists and musicians regularly encounter in their careers. The intense pressure and scrutiny involved in the competition setting, though beyond the scope of this research, allow researchers to observe how different motivational factors may predict performers’ behavior and performance outcomes under stressful conditions. Understanding how these motivations manifest in competitive settings can offer valuable insights into the psychological processes underlying high performance and achievement.

Moreover, the Cliburn Competition’s multi-dimensional structure, encompassing junior, professional, and amateur editions, provides with diverse contexts to explore motivational profiles across various stages of pianists’ development and levels of expertise. By comparing and contrasting the motivational dynamics of emerging talents, seasoned professionals, and enthusiastic amateurs, I can gain a comprehensive understanding of the factors that drive individuals to pursue excellence in piano performance across different career stages.

The junior edition of the Cliburn Competition provides insights into the early developmental stages of high-performing pianists’ careers, offering valuable opportunities to examine how motivational factors influence the trajectories of emerging talents. Given its role as a pinnacle event in the classical music world, the professional edition of the Cliburn Competition serves as a particularly compelling research context. Winners of the competition often go on to enjoy international recognition and prestigious concert engagements, making it an ideal setting to explore the motivational factors that contribute to long-term success and sustained excellence in piano performance. Finally, the amateur edition of the Cliburn Competition offers a unique perspective on the motivational profiles of non-professional pianists, who bring their own diverse array of motivations and goals to the competition. By studying the motivational dynamics of these enthusiastic amateurs, I can gain insights into the distinctive motivational factors that drive individuals to pursue the piano, even outside their professional realms.^2^

### 2.2 Textual analyses on Cliburn competitors’ biographies

In this study, I utilized the Linguistic Inquiry and Word Count (LIWC) software (Pennebaker et al., 2015) to analyze the implicit motives of 107 pianists (juniors: *n* = 38, including 24 competitors and 14 festival artists; professionals: *n* = 30; amateurs: *n* = 39^3^) participating in the 2022–2023 Van Cliburn piano competitions. Operating an internal default word dictionary to quantify the frequency of words within a given text file, the LIWC can be used to assess a wide range of psychological constructs, including “beliefs, fears, thinking patterns, social relationships, and personalities” (Pennebaker et al., 2015, p. 1). Previous research (e.g., Schultheiss, 2013) has demonstrated the utility of LIWC in detecting implicit motives through the analysis of word frequencies in text.

To ensure the replicability of my LIWC analysis, I obtained all biographies from the “Competitors” pages of the Cliburn official website,^4^ retaining only the main biographical text. I removed content such as repertoire^5^ (listed for all competitors), occasional mentions of prizes (e.g., “Bernice Gressman Meyerson First Prize Winner” in S. Hong’s biography, specific to junior competitors), and stand-alone occupational titles not presented within the main biographical text (e.g., “President and CEO – Advisory Investment Management” in E. Santiago’s biography, specific to amateur competitors). This pre-processing step ensures that irrelevant textual data, which could unintentionally confound the frequencies of the “big three” motives of interest, is removed. Critically, the LIWC calculates word frequencies as percentages of the total word count in each text passage, allowing for standardized comparisons across texts despite significant differences in the average word counts of biographies between the three competitor cohorts (junior: *M* = 372.05, *SD* = 66.83; professional: *M* = 281.47, *SD* = 104.63; amateur: *M* = 151.69, *SD* = 19.78), *F*(2, 104) = 98.66, *p* < 0.001.

I employed LIWC2015 (Pennebaker et al., 2015), which includes a default dictionary consisting of 41 categories of psychological constructs, such as drives, affect, and cognition. I focused specifically on the “drives” category, which encompasses the “big three” motives, namely, achievement, power and affiliation. Example words from each category include “win,” “success,” and “better” for achievement; “superior” and “control” for power; and “ally,” “friend,” and “social” for affiliation (Pennebaker et al., 2015, p. 11). The LIWC2015 manual also provides base rates for word usage across various text types (e.g., blogs, novels, Twitter), based on over 231 million words from more than 80,000 writers and speakers.

## 3 Results

### 3.1 Pianists vs. non-pianists

RQ1 examined how the implicit motives of high-performing Cliburn pianists compare to those of non-pianists. The LIWC2015 manual reports the base rates (in %s) for achievement, power, and affiliation motives range from 0.91 to 1.82 (*M* = 1.30, *SD* = 0.82), 1.72 to 3.62 (*M* = 2.35, *SD* = 1.12), and 1.39 to 2.53 (*M* = 2.05, *SD* = 1.28), respectively (Pennebaker et al., 2015, p. 11). In this study, I compared the motive scores extracted from the Cliburn biographies relative to these base rates. The results from a series of independent samples t-tests reveal notable differences between Cliburn pianists across all competition categories (i.e., Junior, Professional, and Amateur) and non-pianists in implicit motives.

**Figure 1:**
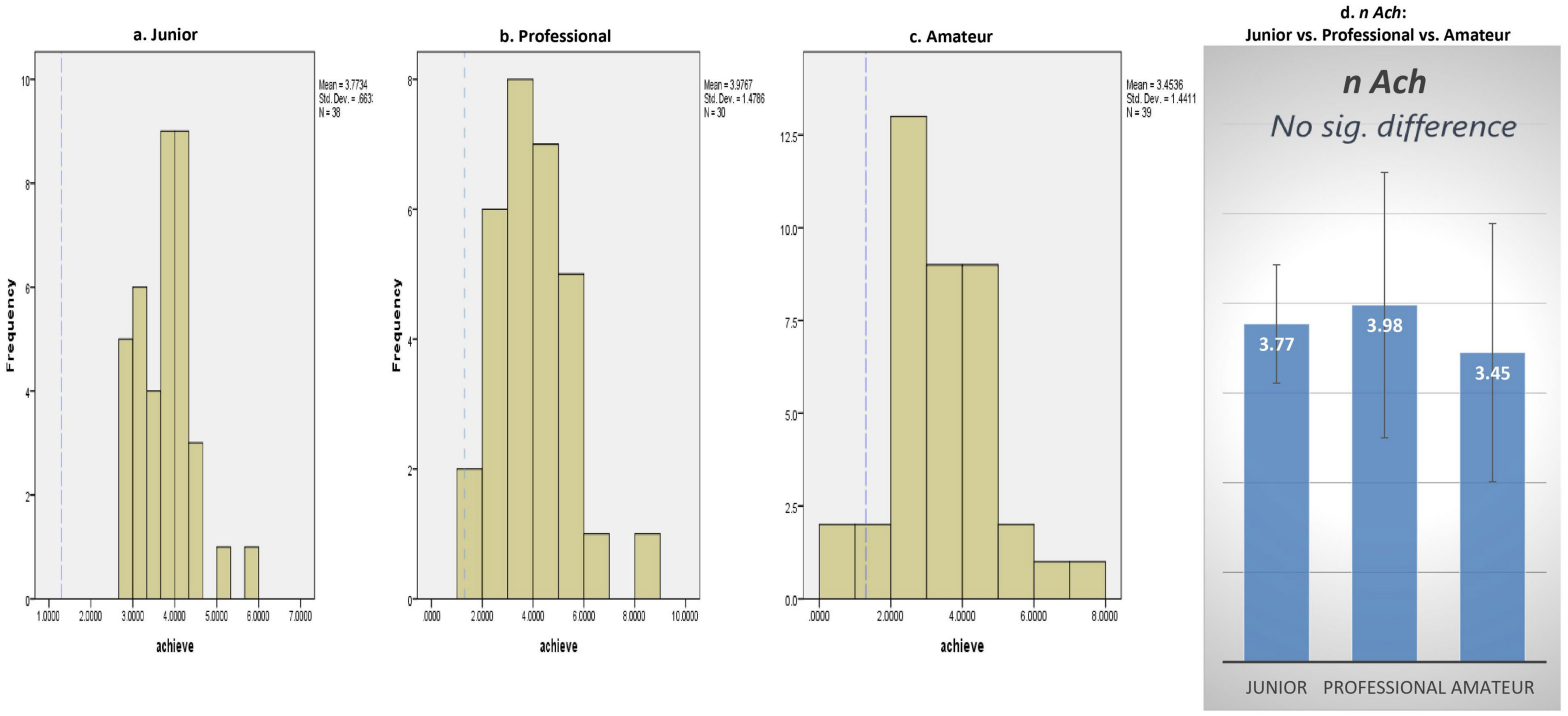
Need for achievement.Note: Blue dotted line in **(a–c)** denotes a grand mean of 1.30 for achievement motive **(d)** is of no concern (Pennebaker et al., 2015).

With regard to *n* Achievement, compared to non-pianists, Cliburn pianists displayed notably elevated levels across all competition categories (Junior: *M* = 3.77, *SD* = 0.66, *t*(37) = 22.99, *p* = 0.000; Professional: *M* = 3.98, *SD* = 1.47, *t*(29) = 9.92, *p* = 0.000; Amateur: *M* = 3.45, *SD* = 1.44, *t*(38) = 9.33, *p* = 0.000) (see Figures 1a–c).

**Figure 2:**
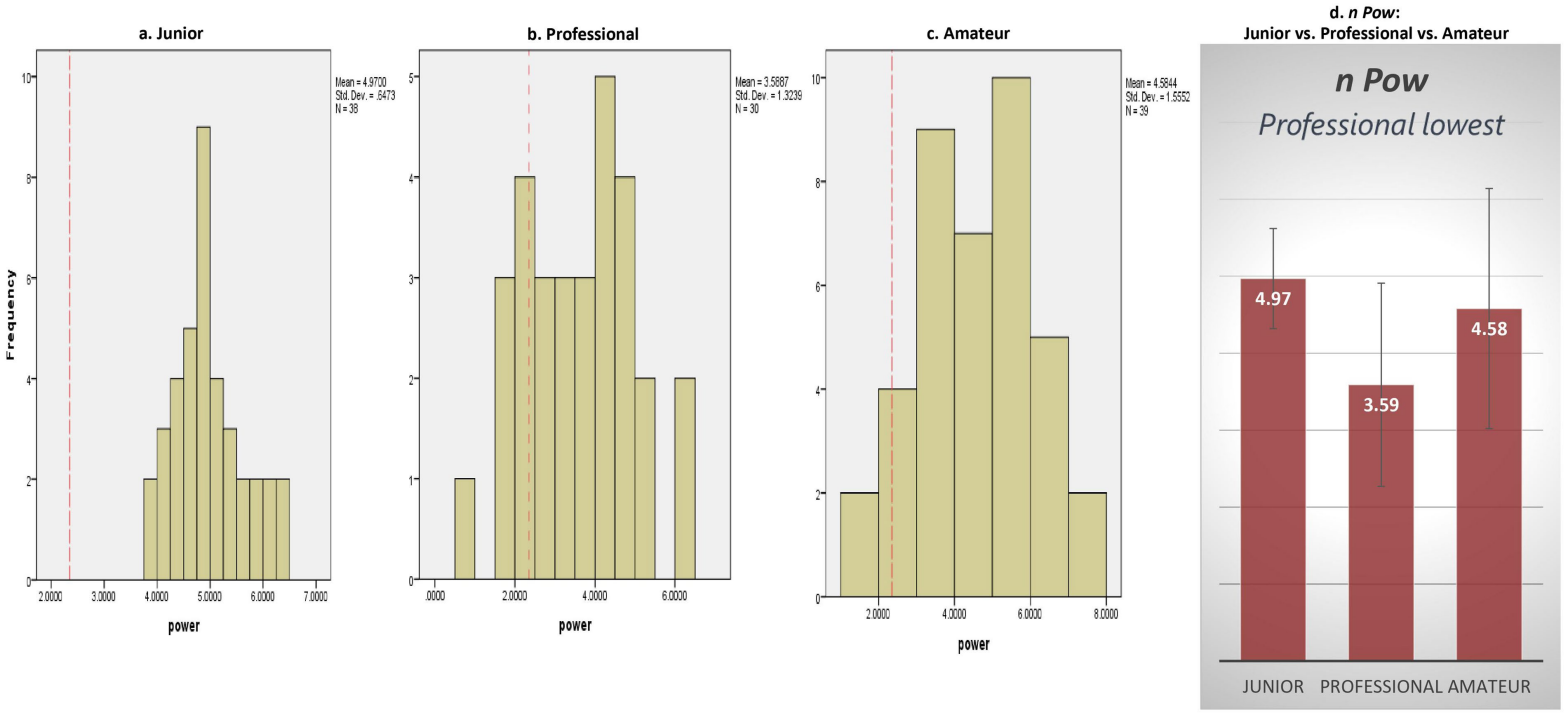
Need for power.Note: Red dotted line in **(a–c)** denotes a grand mean of 2.35 for power motive **(d)** is of no concern (Pennebaker et al., 2015).

Regarding *n* Power, Cliburn pianists demonstrated significantly higher levels across all categories (Junior: *M* = 4.97, *SD* = 0.65, *t*(37) = 24.95, *p* = 0.000; Professional: *M* = 3.59, *SD* = 1.32, *t*(29) = 5.12, *p* = 0.000; Amateur: *M* = 4.58, *SD* = 1.56, *t*(38) = 8.97, *p* = 0.000) (see Figures 2a–c).

**Figure 3:**
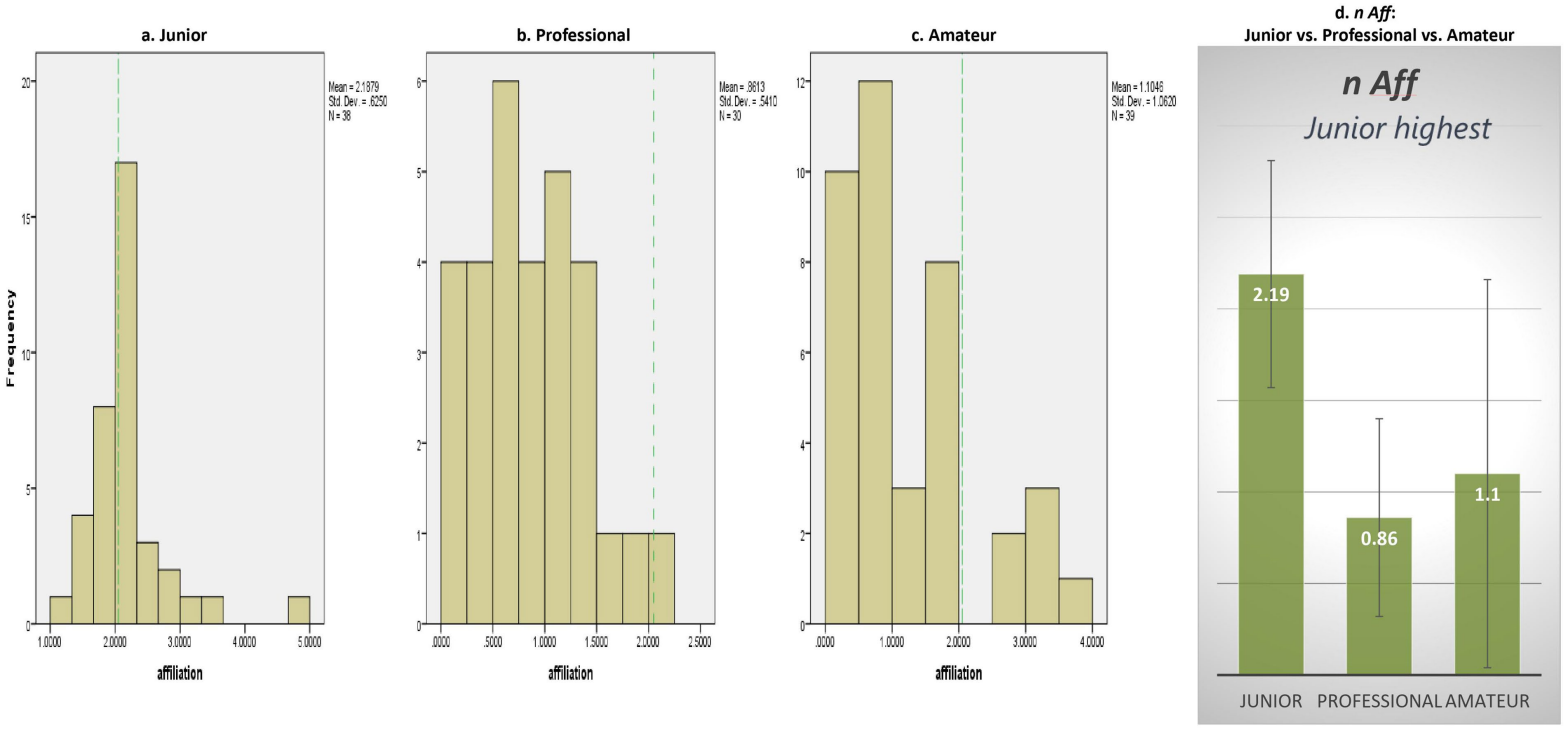
Need for affiliation.Note: Green dotted line in **(a–c)** denotes a grand mean of 2.05 for affiliation motive **(d)** is of no concern (Pennebaker et al., 2015).

However, in the realm of *n* Affiliation, there was no significant difference observed between Junior pianists (*M* = 2.19, *SD* = 0.63) and non-pianists, *t*(37) = 1.36, *n.s.* (see Figure 3a). Both professional [*M* = 0.86, *SD* = 0.54, *t*(29) = −12.03, *p* = 0.000] and amateur pianists (*M* = 1.10, *SD* = 1.06, *t*(38) = −5.56, *p* = 0.000) exhibited significantly lower *n* Affiliation scores than non-pianists (see Figures 3b,c).

Therefore, compared to non-pianists, Cliburn pianists across all competition cohorts demonstrated heightened levels of *n* Achievement and *n* Power. However, unlike junior pianists, adult pianists, regardless of their professional status, were associated with diminished *n* Affiliation.

### 3.2 Junior vs. professional vs. amateur

RQ2 explored whether the implicit motives of high-performing pianists vary across different competitor categories. The findings revealed no significant differences in *n* Achievement across competitor categories, *F* (2, 104) = 1.59, *p* = 0.21 (see Figure 1d).

A significant effect of competitor category was observed on *n Power*, *F* (2, 104) = 10.92, *p* = 0.00 (see Figure 2d). Specifically, professional pianists (*M* = 3.59, *SD* = 1.32) displayed significantly lower *n* Power compared to both amateur pianists (*M* = 4.58, *SD* = 1.56) (*p* = 0.003, *d* = − 0.68) and junior pianists (*M* = 4.97, *SD* = 0.65) (*p* = 0.00, *d* = −1.46). No other differences in *n Power* across competitor categories were found, indicating that professional pianists displayed the *lowest* level of *n* Power.

Moreover, a significant effect of competitor category was observed on *n* Affiliation, *F*(2, 104) = 28.17, *p* = 0.00 (see Figure 3d). Specifically, junior pianists (*M* = 2.19, *SD* = 0.63) displayed significantly higher *n* Affiliation compared to both professional (*M* = 0.86, *SD* = 0.54) (*p* = 0.00, *d* = 2.27) and amateur pianists (*M* = 1.10, *SD* = 1.06) (*p* = 0.00, *d* = 1.29). No other differences in *n* Affiliation across competitor categories were found, indicating that junior pianists demonstrated the *highest* level of *n* Affiliation.

### 3.3 Gender and age

RQ3 explored the relationship between pianists’ sociodemographic variables, such as gender and age, and their implicit motives. Regarding *n* Achievement, male pianists (*n* = 80, *M* = 3.89, *SD* = 1.27) scored significantly higher than female pianists (*n* = 27, *M* = 3.20, *SD* = 0.99), *F*(1, 105) = 6.58, *p* = 0.012. The regression model, which included both age and gender as predictors, was significant, *F*(2, 104) = 3.54, *p* = 0.033, explaining 6.4% of the variance in *n* Achievement. Gender was a significant predictor [*β* = −0.25, *t*(104) = −2.56, *p* = 0.012], while age was not [*β* = −0.13, *t*(104) = −1.34, *n.s.*].

For *n* Power, neither age [*β* = −0.040, *t*(104) = −0.41, *n.s.*] nor gender [*β* = 0.011, *t*(104) = 0.12, *n.s.*] were significant predictors, and the overall model was non-significant, *F*(2, 104) = 0.08, *n.s.* This indicates that neither demographic factor contributed meaningfully to variations in *n* Power.

In terms of *n* Affiliation, age was a significant predictor [*β* = −0.311, *t*(104) = −3.35, *p* = 0.001], with increasing age associated with a decrease in *n* Affiliation. Gender, however, was not a significant predictor for *n* Affiliation [*β* = 0.115, *t*(104) = 1.23, *n.s.*]. The overall model for *n* Affiliation explained 10.6% of the variance, *F*(2, 104) = 6.17, *p* = 0.003.

These findings suggest that gender significantly predicts *n* Achievement, with males scoring higher than females, while age significantly predicts *n* Affiliation, with older pianists showing lower levels of affiliation motivation. Neither age nor gender significantly predicted *n* Power.

## 4 Discussion

### 4.1 Need for achievement

The present findings indicate distinct motivational profiles between pianists and non-pianists. Specifically, compared to non-pianists, pianists demonstrate significantly higher levels of *n* Achievement, indicating a stronger drive for mastery, accomplishment, and success in their musical endeavors. This heightened *n* Achievement among pianists underscores their dedication and persistence in mastering their craft, consistent with the demands of rigorous piano training and performance (Ericsson et al., 1993).

My analysis uncovers gender differences *only* in *n* Achievement among pianists. While both male and female pianists exhibit high levels of *n* Achievement, males tend to demonstrate higher levels compared to females. Although Drescher and Schultheiss’ (2016) meta-analytic evidence showed that men and women did *not* differ in their *n* Achievement (*N* = 2,235, *k* = 13, *d*^∗^ = 0.14, 95%*CI* = [−0.03; 0.30]), the present finding suggests that male pianists may be particularly driven by the pursuit of mastery and accomplishment in their musical pursuits, contributing to a more nuanced understanding of gender dynamics in the context of pianistic motivation.

Moreover, I found that age did not predict *n* Achievement, contrasting Denzinger et al.’s (2016) result that higher age corresponded with lower *n* Achievement (*n* = 736 adults aged 20–80 year). This result suggests that pianists’ drive for personal excellence, artistic mastery, and goal accomplishment remains strong throughout their lifespan and careers. Unlike other broader professional domains where *n* Achievement might decline with age as career goals shift or diminish, classical music emphasizes lifelong pursuit of artistic perfection, where even seasoned musicians strive for continued improvement and artistic growth (Ericsson et al., 1993).

### 4.2 Need for power

This study reveals that pianists also exhibit significantly higher levels of *n* Power compared to non-pianists. This suggests that pianists are more assertive, ambitious, and inclined towards *influencer* roles within their musical communities. The elevated *n* Power among pianists reflects their willingness to assert themselves and strive for opportunities in the highly competitive classical music landscape. However, the study shows that neither age nor gender predicted *n* Power. Drescher and Schultheiss’ (2016) meta-analysis shows that men and women do *not* differ in *n* Power between men and women (*N* = 2,493, *k* = 15, d* = −0.19, 95*CI* = [−0.44, 0.05]). Consistent with this finding, the present results reflect the pianistic domain where individual mastery and aesthetic expression are central, and thus pianists’ desire for influence is *not* differentially shaped by gendered social roles. However, in contrast to Denzinger et al.’s (2016) finding that age negatively correlated with n Power, the present results show that age was not a significant predictor of *n* Power. This discrepancy may suggest that pianists’ desire to influence others does not diminish with age as it might in other fields. Evan as they age, classical pianists’ desire for performance impact and external recognition remains strong throughout their careers. The sustained *n* Power could be tied to the nature of the classical music profession, where personal influence and artistic legacy often grow with experience and artistic maturity (*cf.* Ericsson et al., 1993).

### 4.3 Need for affiliation

The analysis of *n* Affiliation among pianists yields nuanced results. While adult pianists, both professional and amateur, demonstrate significantly lower levels of *n* Affiliation compared to non-pianists, there is no significant difference observed among junior pianists (relative to non-pianists). Moreover, I found that competitors’ age was a significant, negative predictor of *n* Affiliation. These results suggest that the social orientation of pianists may evolve with age and experience, with adults prioritizing individual achievement over social affiliation, whereas juniors may place a greater emphasis on social connections and collaborative experiences in their developmental stage.

In addition, I found that gender was not a predictor of *n* Affiliation, contrasting earlier findings that females typically score higher than males on this motive (Denzinger et al., 2016; Drescher and Schultheiss, 2016). This discrepancy may suggest that the gender differences in *n* Affiliation observed in broader populations may not manifest as strongly within the domain of classical pianists. Given that the piano career often requires networking and collaboration, both male and female pianists might experience similar levels of need for social connection and approval from audiences, peers, and mentors. The focus on individual performance and the shared pursuit of artistic excellence may minimize the typical gender-based differences in *n* Affiliation.

### 4.4 Toward a construct of musician motivational pattern (MMP)

This study reveals distinct musician motivational patterns (MMP) among different Cliburn pianist cohorts (junior, professional, and amateur). Juniors exhibit high levels in all three implicit motives. Professionals demonstrate high levels in *n* Achievement, alongside low levels in both *n* Power and *n* Affiliation. On the other hand, amateurs display high levels in *n* Achievement and *n* Power, while exhibiting low levels in *n* Affiliation. In essence, adult pianists, both professional and amateur, share a similar MMP characterized by high *n* Achievement and low *n* Affiliation, while *n* Power serves as a distinguishing factor between professionals and amateurs, with professionals (amateurs) showing lower (higher) *n* Power. Overall, the MMP emphasizes the primacy of *n* Achievement across pianist cohorts, with *n* Power less prevalent among professionals and *n* Affiliation more prominent among juniors.

## 5 Theoretical and practical implications

Theoretically, the study’s contributions to our understanding of implicit motives in the classical music world can inform broader discussions around the role of motivation and individual differences in *competitive* professional contexts. The major theoretical contributions of this study are at least four-folds: (1) providing insights into the implicit motives of pianists relative to those of non-pianists, (2) delineating the musicianship motivational patterns of pianists in different competing settings and levels (juniors, professionals, amateurs), (3) documenting the association between demographic variables such as gender and age and implicit motives, and (4) offering valuable insights into how pianists can be better supported and motivated in their professional endeavors based on their individual implicit motives and developmental stages. A recent review (Denzinger and Brandstatter, 2018) suggests that implicit motives adapt to life circumstances. The present findings may inform future research on “the trainability of implicit motives” (Denzinger and Brandstatter, 2018) and its associated processes and outcomes both within and beyond the context of classical musicians.

Practically, by understanding the unique motivational patterns of pianists and musician at large, scientist-practitioners can tailor interventions and support strategies to enhance their performance, promote their development, well-being, and success in the dynamic world of classical music. Identifying the implicit motives of pianists at different levels can inform the development of targeted motivational strategies for individuals, tailored to their unique needs and desires. Music educators can tailor their pedagogical strategies to align with a student’s dominant motivational patterns (*cf.* McClelland, 1987; Schultheiss and Brunstein, 2010). For example, pianists high in *n* Achievement are likely to thrive in structured environments with clear goals, regular feedback, and challenges focused on refining technical mastery. In contrast, pianists with higher *n* Affiliation may benefit from collaborative settings (e.g., ensembles, collaborative piano), where interpersonal connections foster their motivation. However, when a particular motivational pattern becomes counterproductive, educators can help students reframe their motivations. For instance, a student driven by *n* Power might focus excessively on competition or external validation, hindering their creativity. In such cases, educators can guide them toward intrinsic rewards, such as artistic expression, personal growth, and the joy of learning, rather than seeking extrinsic recognition (see “Intrinsic motivation principles of creativity” in Amabile, 1996). Still, as an individual’s motivational profile reflects an *organic* combination of the “big three” motives, interventions should not be overly *mechanistic*. When designing support strategies for musicians, it is important to consider how these motives, along with others unexamined (such as the “aesthetic motive” ^6^ discussed below), may interact to shape artistic processes and behavior. This understanding can inform a more holistic and systematic approach to interventions.

## 6 Limitations and future research directions

While this research illuminates the motivational patterns in high-performing classical pianists, several limitations warrant attention. Firstly, the focus of this study was on exploring the implicit motives of pianists participating in the junior, professional, and amateur editions of the 2022–2023 Cliburn competitions. Theoretically, the sole reliance on McClelland’s (1987) “big three” motives may not encompass other significant drives, such as an aesthetic motive central to the artistic pursuits of classical performers like Arturo Benedetti Michelangeli whose obsessive perfectionism in the pursuit of sound quality may be motivated by a deep commitment to artistic ideals that go beyond the “big three.” Future research should incorporate broader motivational frameworks that include aesthetic dimensions to allow for a more nuanced understanding of the motivational landscape and aesthetic process in musicianship (e.g., Cupchik and László, 1992; Christensen, 2018; Juslin and Sloboda, 2010). A promising avenue for future research would be to test and extend Amabile’s (1996) “intrinsic motivation principles of creativity” in the context of musical pursuits. Empirically, the current sample from the Cliburn competitions does not fully represent the diversity of pianists worldwide, underscoring the importance of future research incorporating broader samples. Nonetheless, this sampling approach enables the current research to take a rare initial step in analyzing the motivational dynamics of musical achievement in high-stakes competitive environments. Future studies examining motivational profiles across different instrumentalists and performance contexts could offer a more comprehensive understanding of motivational dynamics in musical performance and achievement. A pertinent question for future research could inquire: *What constitutes the musicianship motivational pattern across* var*ious performance contexts?*

Moreover, the current study employed a typological approach, utilizing dichotomous configurations of motives (e.g., high vs. low *n* Power). Steinmann et al. (2015) employed a dimensional approach through regression analysis with interaction terms to investigate whether the leadership motive pattern (i.e., high *n* Power, low *n* Affiliation, and high AI) predicts managerial performance. Future research could adopt this dimensional approach to examine the relationships between various motive patterns and psychological processes, affective and motivational states, and musician outcomes (*cf.* Chen and Yu, 2016). This prompts a fundamental research question: *How do motivational patterns, or combinations of implicit motives, relate to musicians’ psychological processes and outcomes, and ultimately, their pursuit of musical excellence and fulfillment?*

A third limitation of this research is its focus solely on establishing the association between gender, age, and the implicit motives of pianists, leaving the roles of other critical sociodemographic variables in implicit motives unexplored. Given that piano pursuit is a global phenomenon involving diverse populations, further research is warranted to investigate the interaction between implicit motives and cultural contexts, shedding light on the intricate interplay between internal psychological drives and external socio-cultural factors influencing pianists’ motivation and goal pursuits. Future studies could examine how these motivational patterns interact with societal expectations and cultural norms to shape musicians’ experiences, career trajectories, and professional and personal outcomes. Additionally, comparative research across different cultural contexts could elucidate variations in motivational dynamics across societies and inform culturally sensitive approaches to music education and performance. While implicit motives are individual-level variables, music education and performance are collective endeavors (*cf.* Chen, 2007). Therefore, future research could explore: *How do collective artistic pursuits emerge from the cultivation of individuals’ desires and motives?*

Lastly, a fourth limitation lies in the reliance on cross-sectional data collected within the 2022–2023 Cliburn competitions, which precludes causal inference and limits our ability to assess temporal relationships between motivational factors and pianist outcomes. Future longitudinal designs could offer valuable insights into the developmental trajectories of pianists’ motivations, career paths, and well-being, thereby providing a deeper understanding of the factors influencing long-term success in the classical music industry. Another promising research avenue is to address the causal effects of implicit motives through experiments involving the technique of priming(e.g., Bargh, 1994, 2006). Priming involves subtly influencing individuals’ behaviors, thoughts, or decisions by exposing them to certain stimuli or cues (e.g., photographs, Chen and Latham, 2014) without their conscious awareness of the influence (Bargh and Chartrand, 1999). This technique has been widely employed to study and modify behaviors, including motivation and performance (e.g., Dijksterhuis and Van Knippenberg, 1998; Hart and Albarracín, 2009). Past studies have demonstrated the causal effects of priming on need for achievement (e.g., Chen, 2012; Chen and Latham, 2014; Hart and Albarracín, 2009; Shantz and Latham, 2009). More recent meta-analytic reviews (e.g., Chen et al., 2021; Latham et al., 2023) have established the causal effects of priming goals in the subconscious on both need for achievement and human performance. Future research in the realm of musicianship motivational pattern can leverage this new knowledge to inform the design, development, testing, and validating of effective motive interventions and motivational support systems aimed at fostering musicians’ behavior, performance, and personal and professional development.

In summary, by tackling these research limitations, future research hold promise for advancing the comprehension of the intricate interplay among implicit motives, individual differences, cultural influences, interventional techniques, and performance outcomes across a broader spectrum of performing professionals beyond classical pianists.

## Data Availability

The raw data supporting the conclusions of this article will be made available by the authors, without undue reservation.

## Footnotes

^1^ https://www.carnegiehall.org/About/History/Performance-History-Search?q=yuja%20wang%20philadelphia&dex=prod_PHS&event=68959

^2^My insights into pianists’ motivational patterns have been informed by my own experiences as an amateur pianist and a management scholar in the field of subconscious motivation, particularly through my recent participation in and reflection on the 8th Cliburn International Amateur Competition (2022) and PianoTexas International Festival & Academy (2022 and 2023).

^3^The 2022 Cliburn amateur competition also included 15 additional pianists who participated as non-competing festival participants, but no biographies were provided on the Cliburn website.

^4^Biographies of junior, professional and amateur competitors were obtained from https://cliburn.org/2023-junior-competitors, https://cliburn.org/2022-competitors, and https://cliburn.org/2022-amateur-competitors-3, respectively (all retrievable as of September 25, 2024).

^5^The repertoire choices of pianists during competitions, along with factors such as judges’ evaluations, performance quality, and performance contexts, could significantly influence competition outcomes. However, these factors fall beyond the scope of the current analysis and warrant future empirical investigation.

^6^The author thanks Reviewer 2 for their theoretical insights into the role of an aesthetic motive in the pianistic realm.
